# Biaxial Electrospinning of Poly(lactic acid)/Poly(butylene adipate-co-terephthalate) Blend and Polyvinylpyrrolidone Membranes for Topical Delivery of Betamethasone Valerate

**DOI:** 10.3390/polym18212725

**Published:** 2026-11-02

**Authors:** Édina Liara Schons, Diogo José Salamanca, Gabriel Nardi Fraga, Douglas Cardoso Dragunski, Josiane Caetano

**Affiliations:** 1 Master Program in Chemistry, State University of West Paraná, 645, Rua da Faculdade, Toledo 85903-000, PR, Brazil; edina.schons@gmail.com (É.L.S.); diogo.silva13@unioeste.br (D.J.S.); douglas.dragunski@unioeste.br (D.C.D.); 2 Department of Chemistry, State University of Maringá, 5790, Av. Colombo, Maringá 87020-900, PR, Brazil; gabriel.fraga@unioeste.br

**Keywords:** psoriasis, biaxial electrospinning, PLA, PBAT, topical release

## Abstract

Psoriasis is one of the most common skin diseases in the world, and its treatment results in an improvement in the quality of life of patients, carried out mainly with the use of topical corticosteroids such as ointments and gels. As an alternative to conventional treatment of psoriasis, this work presents a study on the production and characterization of polymeric membranes containing betamethasone valerate produced by biaxial electrospinning. PVP has high solubility in water, resulting in rapid release of the incorporated drug. ECOVIO^®^ (BASF, Ludwigshafen, Germany), composed of PLA and PBAT polymers, has good mechanical properties and forms membranes with controlled drug release, due to its hydrophobic characteristics. The produced membranes were characterized by SEM, FTIR, TGA, DSC and XRD. It was demonstrated that the selected polymers presented adequate conditions of electrospinning for membranes prepared in biaxial mode. Characterization showed a possible compatibility between the drug incorporated in the amorphous state and polymers, which also showed increased thermal stability. An increase in the availability of betamethasone valerate incorporated into the membranes (95%) in relation to the isolated active ingredient (20%) was observed in 24 h. Thus, according to the results obtained, the produced membrane is promising for the controlled release of betamethasone valerate.

## 1. Introduction

Psoriasis is a chronic disease with cutaneous and systemic inflammatory manifestations, non-transmissible, immune-mediated, with genetic predisposition and associated with cardiovascular, psychological, metabolic, and arthritic comorbidities [1].

It affects patients of both sexes and all ages [2], being one of the most common skin diseases [3]. The worldwide prevalence varies between 0.09% and 11.4% [2,4], impacted by variations in age, gender, ethnicity, genetics, environmental, and geographic factors [3].

The most common clinical form of the disease is psoriasis vulgaris, and the traditional clinical manifestation is the presence of erythematous, clearly demarcated, and pruritic plaques covered by silvery scales, affecting approximately 90% of patients [4,5]. Treatment of injuries results in an improvement in the quality of life of patients. Topical treatment of lesions is an option for cases of mild to moderate manifestation of the disease, while for severe cases, topical treatment can be performed in parallel with systemic treatment or as maintenance treatment during disease remission [6].

Emollients in creams and lotions are used for maintenance after the use of corticosteroids [2,7]. The corticosteroids of choice for treatment are clobetasol and dexamethasone, as well as betamethasone and mometasone, which have a similar scope to dexamethasone. Currently available in Brazil for topical treatment are creams, gels, ointments, and hair lotions containing the medications of choice for treatment (betamethasone, dexamethasone, clobetasol, and calcipotriol).

The choice of vehicle used for formulation can significantly alter the use and penetration of the drug into the body, impacting the expected therapeutic effect, in addition to influencing the patient’s adherence to treatment. Occlusive treatment of topical medications (which consists of coating the medication with films) increases the effectiveness of the product [8]. Betamethasone valerate in the pharmaceutical form of a plaster, in transparent occlusive films, has shown significant improvement in psoriatic plaques, to the advantage of using creams and ointments [9].

Nanofibers produced by electrospinning for encapsulating drugs for direct delivery to psoriatic plaques have been one of the options for developing new forms of treatment for the disease. In addition, few studies have been carried out, and there are a variety of possibilities to be made with different polymers, drugs, and types of electrospinning [10].

Nanofibers are thin threads manufactured by physical processes using synthetic chemicals [11]. It presents interesting characteristics such as large surface area, possibility of surface functionalization, adjustable porosity, a wide range of materials that can be used, in addition to favorable mechanical performance. It has several biomedical applications in tissue engineering, wound-healing dressings, biosensors, biomedical devices, and as a drug delivery system [12].

Nanofibers are generally produced by electrospinning as it is relatively simple and economically viable [10]. Electrospinning can be divided into six distinct categories: basic electrospinning, blend electrospinning, coaxial electrospinning, emulsion electrospinning, melt electrospinning, and gas jet electrospinning. Polymer blend electrospinning is efficient for drug delivery systems, while emulsion electrospinning forms core–shell structured nanofibers, which can better protect the encapsulated active from environmental conditions [13]. 

**Figure 1:**
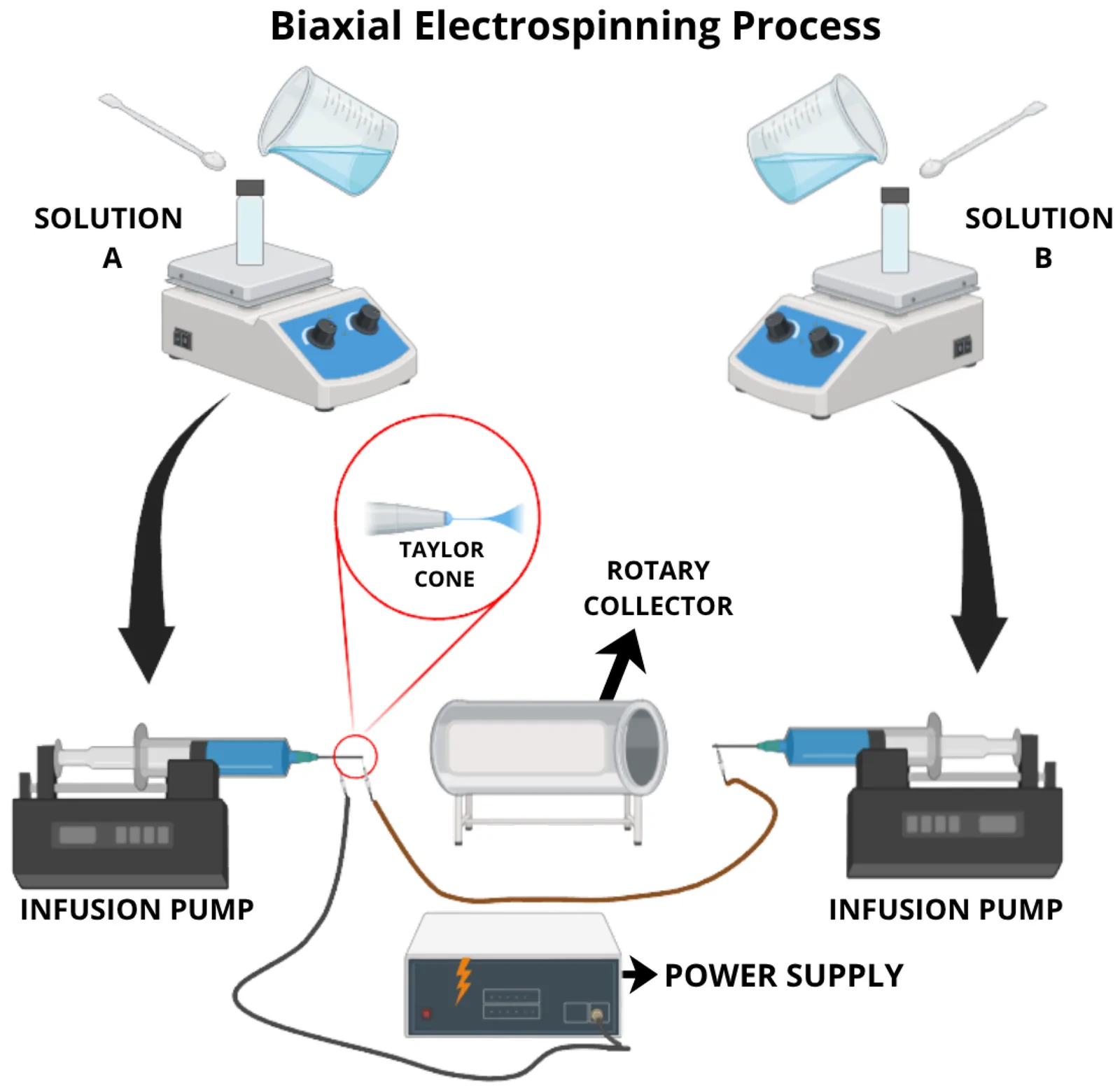
Schematic of the biaxial electrospinning process.

The characteristics of the polymer will predict the release profile of the incorporated drug, based on its chemical, mechanical, and biological properties [14]. Water-soluble polymers (such as PVP) influence the membrane’s water absorption capacity, permeability, and degradability of the material [14]. They are characterized by an initial release of the drug. When mixed with hydrophobic polymers (such as ECOVIO^®^), the degradation of the polymer becomes more controlled due to the decrease in the diffusion rate of the drug through the fiber [15]. Biaxial electrospinning is a technique in which two pumps containing two distinct nozzles with different polymer solutions can be positioned on opposite sides of a rotating collector and electrospun simultaneously [16]. Figure 1 illustrates the biaxial electrospinning process. This method enables the production of a membrane from polymers with different characteristics, depending on the intended application.

Thus, the present work aims to produce an electrospun polymer membrane by a biaxial mode, in which a polymer solution containing the hydrophilic polymer PVP is electrospun for rapid drug release, while the other solution contains the hydrophobic polymer ECOVIO^®^, aiming to promote sustained release. In addition, the physicochemical interaction between the components of the membrane was evaluated, and a preliminary in vitro release study was carried out to assess the release profile of VBM from the produced matrix. It should be noted that this study was limited to drug release under simulated physiological conditions and does not assess skin permeation, skin retention, or therapeutic efficacy.

## 2. Materials and Methods

### 2.1. Materials

Polyvinylpyrrolidone (PVP) (Mw: 360,000 Da) from Sigma-Aldrich (Burlington, MA, USA) and a blend under the trade name ECOVIO^®^ produced by BASF (Ludwigshafen, Germany), composed of 45% poly(lactic acid) (PLA) and 55% poly(butylene adipate-co-terephthalate) (PBAT), were used to produce the electrospun membrane. Chloroform (99.8%), N,N-dimethylformamide (DMF; 99.9%), Ethanol (99.9%), potassium phosphate (98%), and sodium hydroxide were acquired from Neon (Suzano, SP, Brazil). Betamethasone valerate (VBM) was acquired in a local pharmacy (Toledo, PR, Brazil).

### 2.2. Preparation and Electrospinning

Initially, an optimization step was conducted to obtain uniform fibers and the lowest number of beads possible. For this optimization step, three different polymer concentrations were evaluated, as well as electrospinning parameters such as the distance from the needle to the collector (cm), the applied voltage potential (kV), and the solution flow rate (mL h^−1^).

The polymer solution was prepared by dissolving the amount of PVP (5%, 8%, and 10% (w v^−1^)) in ethanol and the amount of ECOVIO^®^ (10%, 15%, and 20% (w v^−1^)) in a mixture of solvents composed of 85% (v v^−1^) chloroform and 15% (v v^−1^) N,N-dimethylformamide. The solutions were magnetically stirred for 24 h, and then 1% (w w^−1^) betamethasone valerate was incorporated into the solution.

The solutions were then transferred to a 10 mL glass syringe containing a Hamilton needle (diameter of 0.7 mm) and electrospun by varying the distance from the needle to the collector (12 and 15 cm), the applied voltage potential (14 and 18 kV), and the solution flow rate (1.0, 1.8, and 2.5 mL h^−1^). The electrospun membranes were collected on aluminum foils fixed on a rotating collector rotating at 200 rpm. The electrospinning and biaxial electrospinning occurred at room temperature and relative air humidity ranging from 30 to 60%.

The optimum conditions to obtain more homogeneous fibers with fewer beads were defined by multi-parameter tests during electrospinning. The conditions used were: solution concentration of 8% (w v^−1^) for PVP and 20% (w v^−1^) for ECOVIO^®^, distance from the needle to the collector of 15 cm, applied voltage potential of 18 kV, and solution flow rate of 1.8 mL h^−1^. After the optimization step, six membranes were produced according to Table 1. Both membranes, PVP/ECOVIO^®^ and PVP/ECOVIO^®^/VBM, were produced by biaxial electrospinning using previously prepared polymeric solutions.

### 2.3. Membrane Characterization

The morphology of the membranes was investigated by scanning electron microscopy (SEM) using QUANTA 250 (Thermo Fisher Scientific, Waltham, MA, USA) equipment, at magnifications ranging from 2,000 to 10,000 times. The samples were placed on a double-sided carbon adhesive tape fixed to a support and metallized with a thin layer of gold to a thickness of 30 nm. The mean fiber diameter of each membrane was determined using the image processing software Imagej 1.54j performing measurements in random parts of random fibers (n = 100). The results are expressed by mean ± standard deviation (SD). Interactions between the polymers and the drug were evaluated in a PerkinElmer Frontier^®^ (Waltham, MA, USA) Fourier transform mid-infrared spectrophotometer (FTIR), using the Attenuated Total Reflectance (ATR) module in the wave number ranging from 4000 to 600 cm^−1^, with a resolution of 2 cm^−1^ and 16 scans. Thermogravimetric analysis was performed on PerkinElmer equipment (model STA6000). Approximately 17 mg of each membrane were cut out and evaluated in the temperature range of 30 to 600 °C, using alumina crucible and heating rate of 10 °C min^−1^ under nitrogen atmosphere (flow rate of 20 mL min^−1^). Differential Scanning Calorimetry (DSC) analysis was performed using Shimadzu (Kyoto, Japan) equipment (model DSC-60). For this technique, approximately 20 mg of each membrane were cut out and evaluated in the temperature range of 30 to 200 °C, with a heating rate of 10 °C min^−1^, under a nitrogen atmosphere (flow rate of 20 mL min^−1^). The crystallinity of the membranes was evaluated by X-ray diffractometry, using Bruker^®^ (Billerica, MA, USA) equipment (model D2 Phaser), at an angle of 2θ, ranging from 5 to 80° with an increment of 0.02° (CuKα radiation source of λ = 1.5418 Å).

### 2.4. In Vitro Release Study

In vitro release studies were conducted by fixing a portion of the PVP/VBM, ECOVIO^®^/VBM, and PVP/ECOVIO^®^/VBM membranes in a proper holder with a diameter of 3.5 cm and immersed in the release medium. The devices containing the PVP/VBM and ECOVIO^®^/VBM membranes were placed in a 100 mL beaker containing 25 mL of medium, whereas the PVP/ECOVIO^®^/VBM membrane was added to 50 mL of medium in a 100 mL beaker. The difference in volume used for each membrane is due to the theoretical amount of drug present in each membrane, ensuring that the maximum VBM concentration falls within the established calibration curve. Release medium was composed of phosphate buffer pH 5.7, which was kept under constant stirring (100 rpm) in a New Lab (Piracicaba, SP, Brazil) orbital shaking incubator at controlled temperature (32 ± 2 °C).

Samples were collected at 2, 5, 10, 15, and 20 min for the PVP/VBM membrane. For the ECOVIO^®^/VBM and PVP/ECOVIO^®^/VBM membranes, samples were collected at 5, 10, 15, 20, 30, 60, 60, 90, 120, 240, 360, and 1440 (24 h) minutes. Aliquots were collected and analyzed in a Shimadzu UV-Visible (UV-1800) at the wavelength of 241 nm. After each collection and reading, the aliquot was returned to the respective release medium to maintain a constant solution volume. The samples were quantified against a calibration curve ranging from 0.5 to 20 mg L^−1^, prepared from a stock solution of VBM in the release medium. Furthermore, to compare the solubility of the drug incorporated into the polymeric membranes with that of the VBM raw material, a drug solubilization study drug was performed under the same conditions to which the membrane was subjected.

The samples were quantified against a five-point calibration curve with concentrations of 0.5, 5.0, 10.0, 15.0, and 20.0 mg L^−1^, prepared from a 50 mg L^−1^ betamethasone valerate stock solution in phosphate buffer (pH 5.7), which can be found in the Supplementary Material (Figures S1 and S2). The calibration curve showed excellent linearity over the concentration range studied (R^2^ = 0.999). Additional calibration data is available in the Supplementary Material (Figures S1 and S2).

For the in vitro release experiments, membrane batches were prepared independently on different production days under the same electrospinning conditions. Release studies were performed in triplicate using samples obtained from these independently prepared batches. The results are presented as mean ± standard deviation (SD), and the error bars shown in the release profiles correspond to the standard deviation of three independent membrane batches.

The experimental conditions employed in this study were designed to evaluate the in vitro release behavior of VBM from the electrospun membranes. Therefore, the test does not reproduce the barrier properties of human or psoriatic skin and should not be interpreted as a permeation or retention study.

The VBM release from the membranes were evaluated using the software DDSolver 1.0 [17] to compare different kinetic models such as zero order, first order, Higuchi, Korsmeyer & Peppas, Hixson-Crowell, and Weibull.

## 3. Results and Discussion

### 3.1. Membrane Characterization

#### 3.1.1. Morphology

**Figure 2:**
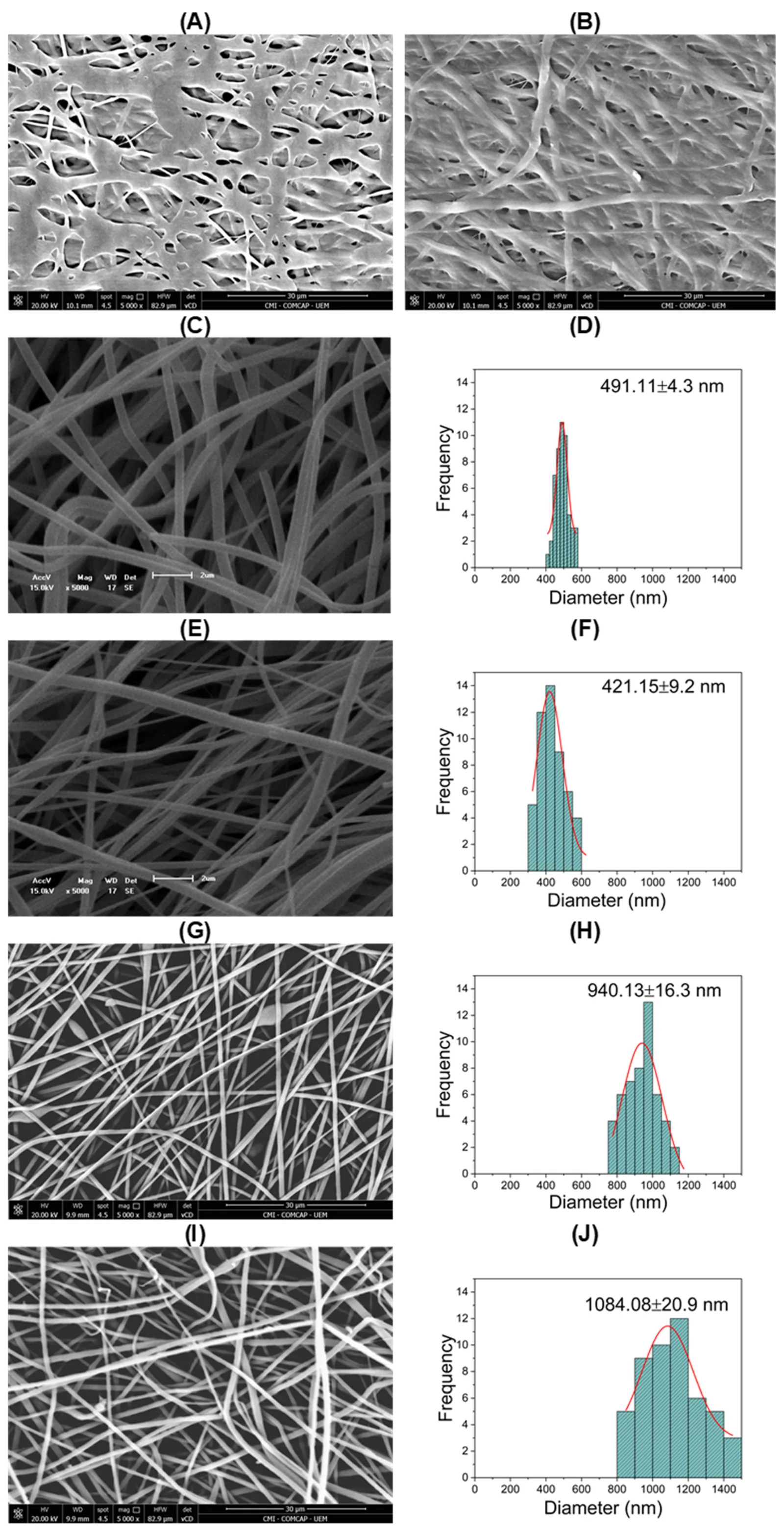

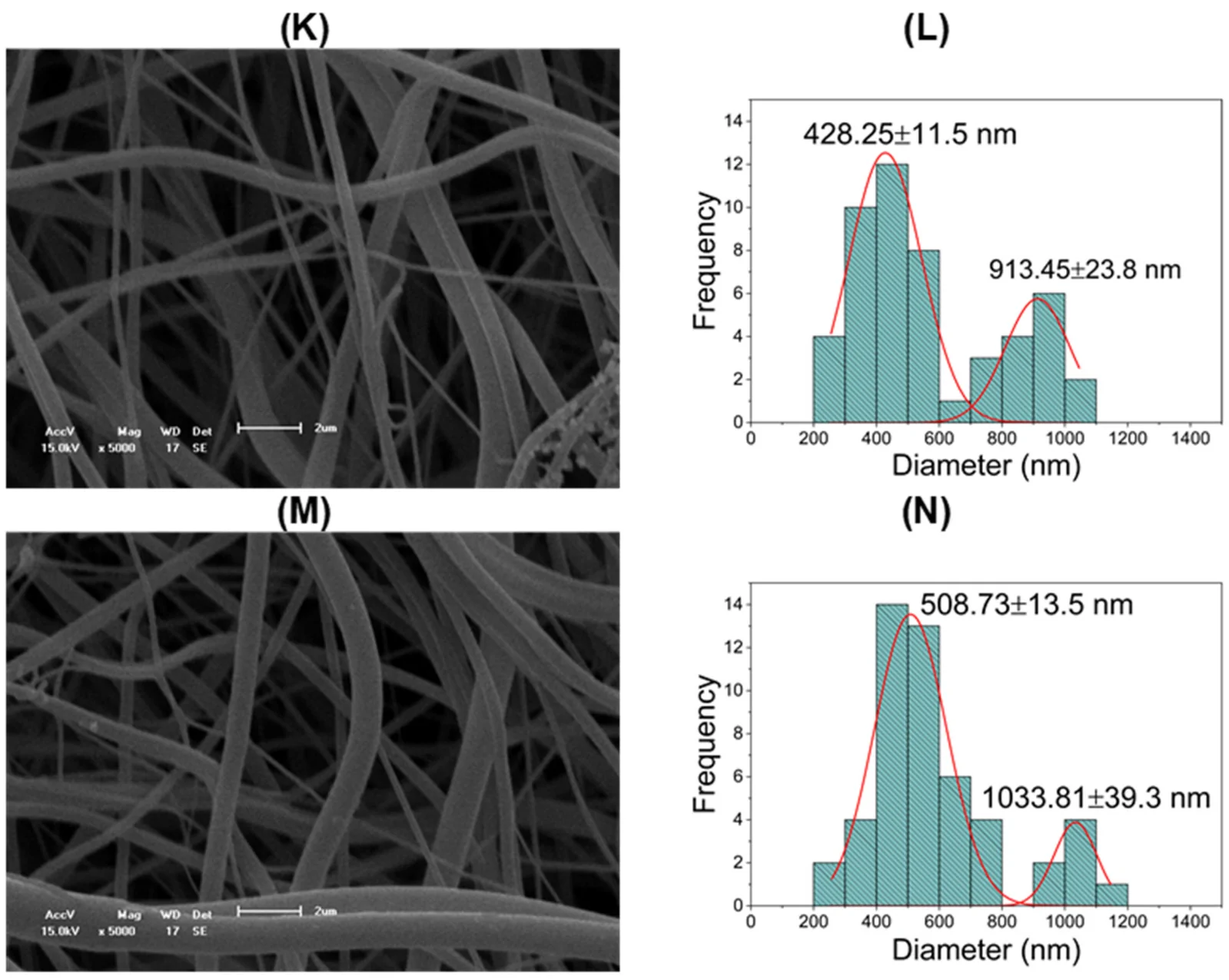
Scanning Electron Microscopy (SEM) at 5000 magnification and the normal distribution plot (n = 100) with results expressed by mean ± standard deviation (SD). (**A**) PVP with coalescence; (**B**) PVP/VBM with coalescence; (**C**) PVP; (**D**) PVP normal distribution plot; (**E**) PVP/VBM; (**F**) PVP/VBM normal distribution plot; (**G**) ECOVIO^®^; (**H**) ECOVIO^®^ normal distribution plot; (**I**) ECOVIO^®^/VBM (**J**) ECOVIO^®^/VBM normal distribution plot; (**K**) PVP/ECOVIO^®^; (**L**) PVP/ECOVIO^®^ normal distribution plot; (**M**) PVP/ECOVIO^®^/VBM; (**N**) PVP/ECOVIO^®^/VBM normal distribution plot.

Morphological analysis was performed using scanning electron microscopy (SEM) to verify whether the electrospun membranes presented uniform fibers without beads and whether the incorporation of the drug affects the morphological characteristics of the membranes. Figure 2 presents the images obtained by SEM analysis of PVP, PVP/VBM, ECOVIO^®^, ECOVIO^®^/VBM, PVP/ECOVIO^®^, and PVP/ECOVIO^®^/VBM membranes and the normal distribution plots of the diameters of the fibers.

Figure 2A,B show the coalescence of the membranes PVP and PVP/VBM after storage. This happens because PVP polymer has hygroscopic characteristics, causing the membrane to absorb moisture from the air, resulting in the deformation of the fibers [18]. For the PVP membranes that did not show coalescence (Figure 2C), a decrease in the average diameter of the fibers from 491.11 nm ± 4.3 nm to 421.15 ± 9.2 nm can be observed when the drug was added (Figure 2D). The decrease in the average diameter of the wires with the addition of VBM may be associated with the interaction between the drug and the polymer that can affect the viscosity of the solution [19]. The main expected interaction that occurs between VBM and PVP is hydrogen bonds between the C = O bond of PVP and the hydroxyl group (−OH) of VBM [18,20].

Electrospinning occurs when electrical forces on the surface of a polymer solution overcome the surface tension and cause an ejection. Solution additives have an impact on the electrospinning process of polymer solutions and the physicochemical properties of the fibers formed [21]. The viscosity of the polymer solution is one of the parameters that affects the electrospinning process and can impact the average diameter of the wires. Lower viscosities tend to form thinner fibers, and as viscosity increases, the process of stretching the polymer jet becomes more difficult, increasing the diameter of the fibers [22].

The ECOVIO^®^ membranes (Figure 2G) present a larger average fiber diameter than those observed for the PVP membranes. For the membrane ECOVIO^®^/VBM, showed in Figure 2I, which contains the incorporation of the drug, it was possible to observe a less homogeneous surface compared to the fibers of the membrane prepared only with the polymer, as well as an increase in the average wire diameter from 940.13 ± 16.3 nm to 1084.08 ± 20.9 nm (Figure 2H,J).

The lower homogeneity, as well as the increase in the average diameter of the fibers, may be associated with the incorporation of the drug into the membranes. Alves et al. also observed an increase in the diameter of electrospun PLA fibers when the VBM was incorporated compared to pure fibers [23]. In the membrane ECOVIO^®^/VBM, it is possible to see some thinner fibers and loose ends due to the rupture of the fibers. This may indicate that the wires became more fragile and friable with the incorporation of VBM. This was also observed by Yu, et al. (2009) in electrospun membranes of PVP and ibuprofen [24]

For membranes prepared in biaxial mode, two fiber size distributions can be observed. For the membrane PVP/ECOVIO^®^ in Figure 2K, the mean diameters obtained (Figure 2L) were 428.25 ± 11.5 µm and 913.45 ± 23.8 µm, and for the membrane (PVP/ECOVIO^®^/VBM), showed in Figure 2M, there was an increase in the mean diameter (Figure 2N) of the two distributions to 508.73 ± 13.5 µm and 1033.81 ± 39.3 µm. The distinct diameter distributions can be associated with each electrospun polymer for membrane formation, with threads with smaller diameters referring to PVP fibers, while the larger ones refer to ECOVIO^®^ fibers. Furthermore, a greater quantity of PVP fibers can be observed in relation to ECOVIO^®^, demonstrating that the distribution of fibers during electrospinning was not homogeneous. As in the other membranes, the membrane containing the drug presented less homogeneous threads and with some rupture points due to a possible interaction with the VBM.

No coalescence was observed in PVP/ECOVIO^®^ membranes prepared in biaxial mode and stored in a similar way to pure PVP fibers. This is due to the presence of ECOVIO^®^, which has hydrophobic characteristics, suggesting that ECOVIO^®^ makes the PVP fibers less susceptible to moisture. Obtaining the membrane using the biaxial technique may also have helped decrease moisture in PVP fibers associated with ECOVIO^®^. In biaxial mode, polymer solutions are electrospun on each side of the rotary collector, and the fibers ejected from the syringes are collected in an interleaved manner. In this way, the PVP fibers are collected between the ECOVIO^®^ threads, possibly becoming more protected from humidity when compared to membranes that contain only PVP, or if the membrane had been produced in the conventional way, applying one polymer solution after another. The SEM images reveal, in general, membranes composed of continuous fibers, without beads, and with a reduced quantity of visible particles, demonstrating that the drugs and the selected polymers present adequate conditions for electrospinning. Furthermore, no drug particles were observed on the surface of the wires, indicating good compatibility between the polymers and the drug or encapsulation of the drug by the polymers. Further moisture-aging experiments, measurements of the contact angle, and water/water vapor uptake are still required to confirm moisture protection provided by ECOVIO^®^ to PVP fibers.

#### 3.1.2. Fourier-Transform Infrared Spectroscopy (FTIR)

**Figure 3:**
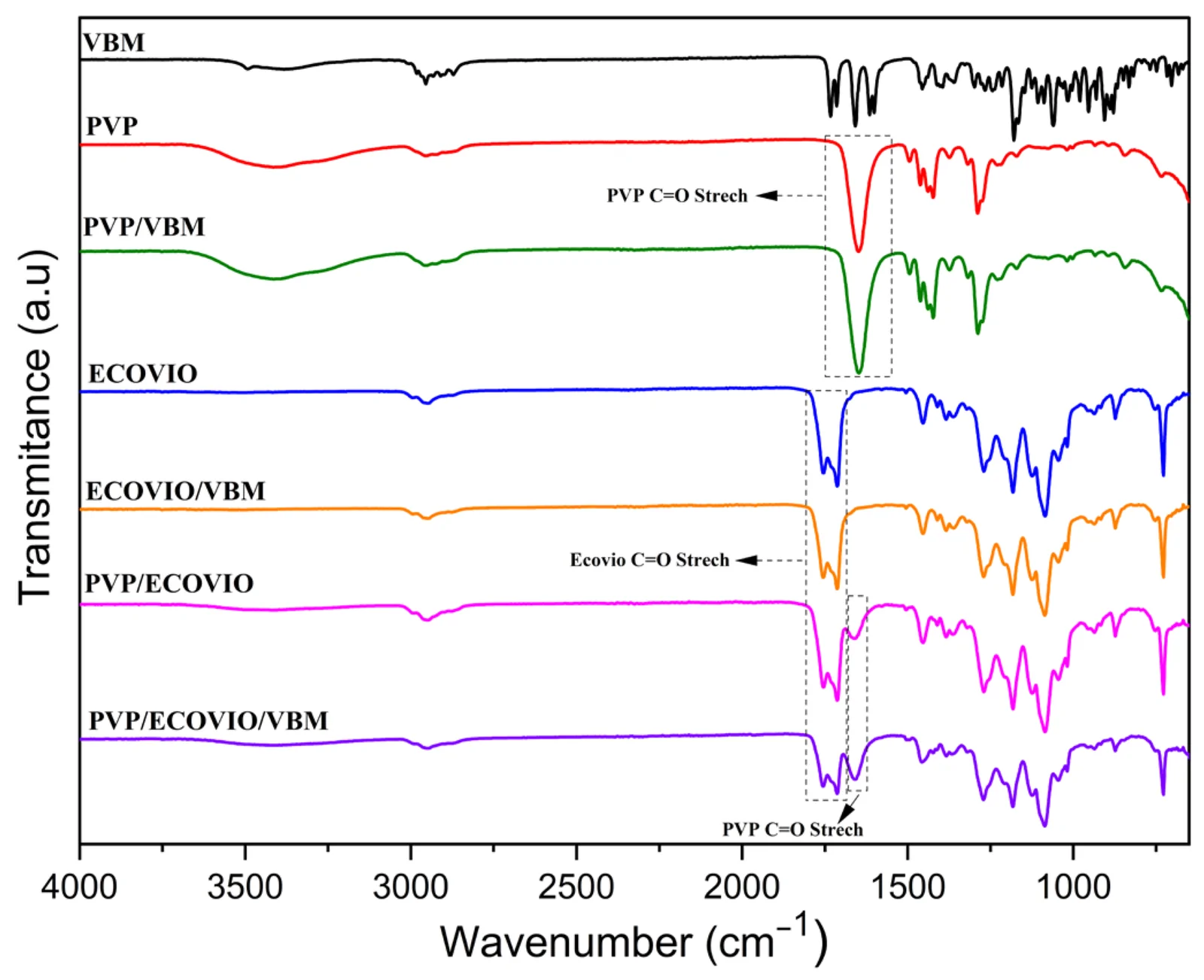
Comparison of FTIR spectra of VBM and membranes PVP, PVP/VBM, ECOVIO^®^, ECOVIO^®^/VBM, PVP/ECOVIO^®^, and PVP/ECOVIO^®^/VBM.

Through mid-infrared spectroscopy, it is possible to observe the chemical interactions and changes in the membrane components by identifying the main functional groups of betamethasone valerate, ECOVIO^®^, and PVP in the acquired FTIR spectra (Figure 3). The PVP/VBM membrane presents the same bands observed in the spectrum of the PVP membrane, while the spectrum of the ECOVIO^®^/VBM membrane presents the same bands at the same wave numbers observed in the spectrum of the ECOVIO^®^ membrane. It is not possible to identify any band shift and no additional band characteristic of the VBM drug. Regarding the membranes PVP/ECOVIO^®^ and PVP/ECOVIO^®^/VBM, a predominance of bands related to ECOVIO^®^ can be observed in the FTIR spectrum obtained. The appearance of the band at 1664 cm^−1^ related to the stretching (C = O) of the PVP carbonyl, the most prominent functional group in the polymer spectrum, stands out [25].

No characteristic band of the polymers showed a shift in the spectrum, and the membrane PVP/ECOVIO^®^/VBM presented a spectrum equal to that of the membrane without the drug PVP/ECOVIO^®^. This demonstrates that the drug shows no chemical incompatibility with the PVP and ECOVIO^®^ polymers since it did not present any alteration or interaction with the main functional groups of the polymers used in the membrane.

The absence of characteristic VBM bands in the membranes may be related to the low concentration of the drug added to the polymer solutions, since only 1% of VBM was incorporated into each polymer. In addition, it is worth noting that the main characteristic functional groups in the drug are also present in the polymers. Thus, due to the high concentration of polymer in relation to the drug, the characteristic bands of the polymers overlap the VBM bands. Another possibility is that during the electrospinning process, the drug remains incorporated inside the polymeric fibers. The technique used to incorporate VBM into the membranes was the mixed electrospinning technique, where the drug is solubilized in the polymeric solution before being electrospun.

VBM has hydrophobic characteristics, as does ECOVIO^®^, so the drug remains completely solubilized in the polymer solution during the electrospinning process, facilitating encapsulation in the wires. PVP has an amphiphilic characteristic. Due to the presence of the amide group in pyrrolidone, PVP has hydrophilic characteristics and hydrophobic characteristics due to the presence of alkyl groups [18]. This characteristic results in a good interaction with both hydrophilic and hydrophobic drugs, which can also lead to the encapsulation of VBM.

#### 3.1.3. Thermogravimetric Analysis (TGA)

**Figure 4:**
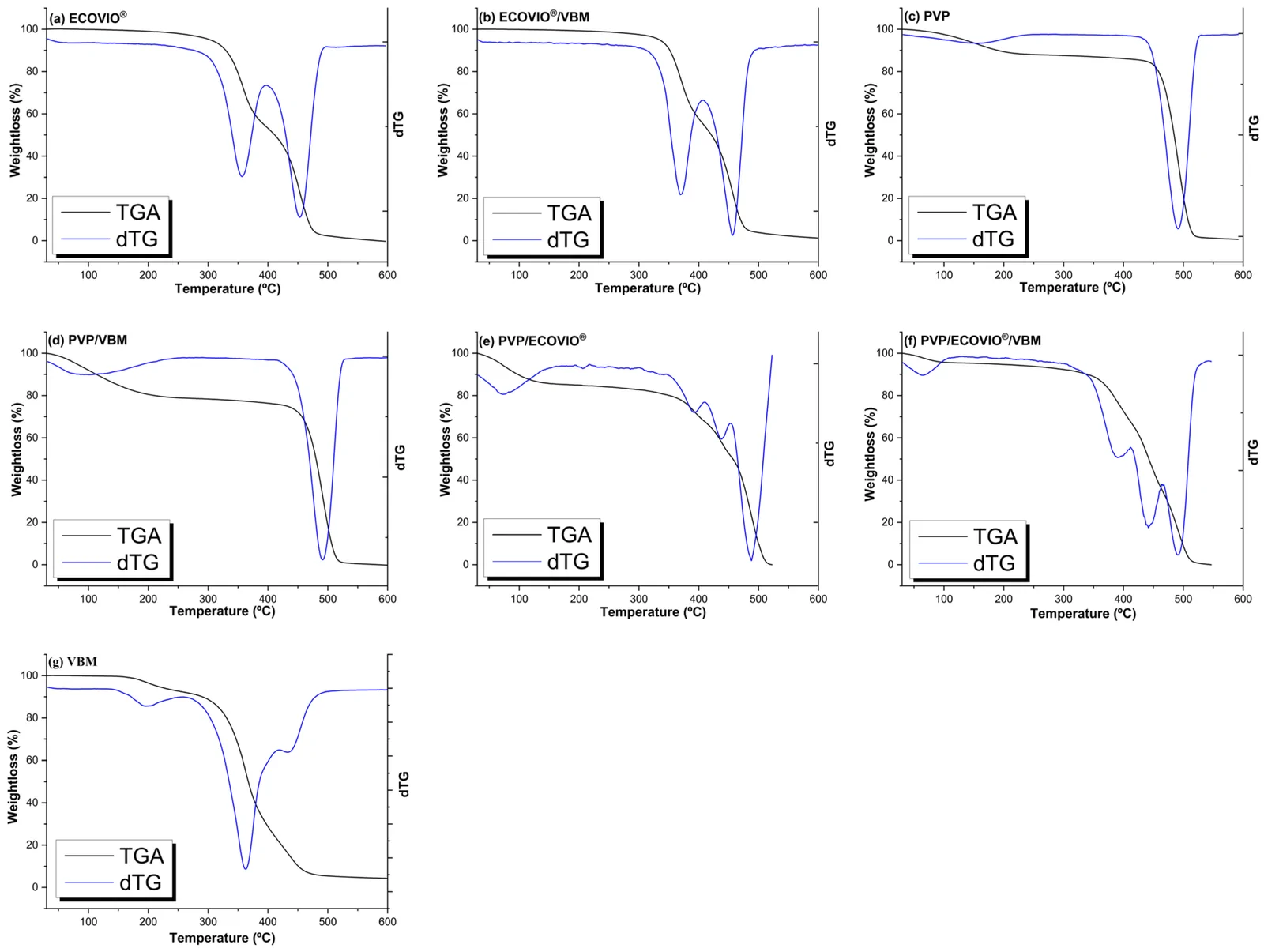
TGA curve and first derivative (dTG) of the membranes: (**a**) ECOVIO^®^; (**b**) ECOVIO^®^/VBM; (**c**) PVP; (**d**) PVP/VBM; (**e**) PVP/ECOVIO^®^; (**f**) PVP/ECOVIO^®^/VBM; (**g**) VBM.

TGA analysis was performed to determine the thermal stability of the electrospun membranes and the impact of drug incorporation and polymer blending in biaxial electrospinning. By comparing the temperatures of mass loss events, especially those related to polymer decomposition, the effect of drug incorporation on the polymers can be understood. Figure 4 shows the thermogravimetric curve (TGA) and the first derivative (dTG) of the VBM raw material (Figure 4g) and the membranes PVP (Figure 4c), PVP/VBM (Figure 4d), ECOVIO^®^ (Figure 4a), ECOVIO/VBM (Figure 4b), PVP/ECOVIO^®^/VBM (Figure 4e), and PVP/ECOVIO^®^ (Figure 4f).

The VBM presented two mass loss events related to three degradation events. The first degradation event occurred between 162 °C and 286 °C with a loss of 7.5% of mass, while the second and third events occurred between 286 °C and 480 °C with a loss of 86.5% of mass, adding up to 94.0% of the total mass of the sample [26].

The membranes PVP and PVP/VBM present mass loss of up to 20% and up to approximately 200 °C, referring to the loss of water adsorbed on the polymer surface due to its hygroscopic characteristic. The second mass loss event of PVP refers to the decomposition of the polymer and presents T°_initial_ at 435 °C and T°_final_ at 520 °C, with loss of approximately 97.9% of the total mass [27]. It can be observed that there is no shift in the temperatures of degradation events in the membrane containing the drug, demonstrating that the incorporation of VBM in the PVP membrane did not present an impact on the thermal stability of the polymer.

The TGA curve for the fiber ECOVIO^®^ shows that there is no significant mass loss up to approximately 286 °C (initial T), where degradation of the blend polymers (PLA and PBAT) begins to be observed. ECOVIO^®^ presents two well-defined events between 286 °C and 470 °C, with mass loss of approximately 95% at 470 °C. The first event, with the maximum mass loss rate shown by the derivative curve (-dTG) at 354 °C, refers to the degradation of PLA, while the event at 453 °C is related to the degradation of PBAT [28]. Similarly, the degradation peaks of PLA and PBAT can be observed in the TGA of the membrane ECOVIO^®^/VBM. A shift in the T°C_initial_ and in the PLA degradation event, 320 °C and 370 °C, respectively, can be observed, indicating a possible interaction between the drug and PLA. However, the incorporation of the drug had no effect on the decomposition temperature of PBAT, which remained at 453 °C, and on the T°C_final_, at 470 °C with a loss of approximately 95% of mass.

In the membrane PVP/ECOVIO^®^, four mass loss events are observed. The first event is related to the loss of approximately 14% of water up to approximately 130 °C. The following events are related to the decomposition of the polymers. The T°C_initial_ of the degradation of PLA increased by 69 °C, reaching 355 °C, while the maximum degradation temperature increased by 38 °C (392 °C). The maximum degradation peak of PBAT decreased by 16 °C, reaching 437 °C. A third event is observed at 488 °C and refers to the degradation of PVP. Thus, the interaction between the polymers is possibly occurring, increasing the thermal stability of the membrane, mainly regarding the stability of the PLA polymer. Regarding the membrane PVP/ECOVIO^®^/VBM, a loss of approximately 4% of water can be observed up to 130 °C. Mass loss related to the degradation of the PLA, PBAT, and PVP polymers can also be observed, with temperatures close to those observed in the membrane PVP/ECOVIO^®^ at 392, 440, and 490 °C, respectively, demonstrating that the incorporation of the drug did not alter the thermal stability of the membrane, since the increase in degradation temperatures is related to the interaction between the PVP and ECOVIO^®^ polymers.

#### 3.1.4. Differential Scanning Calorimetry (DSC)

**Figure 5:**
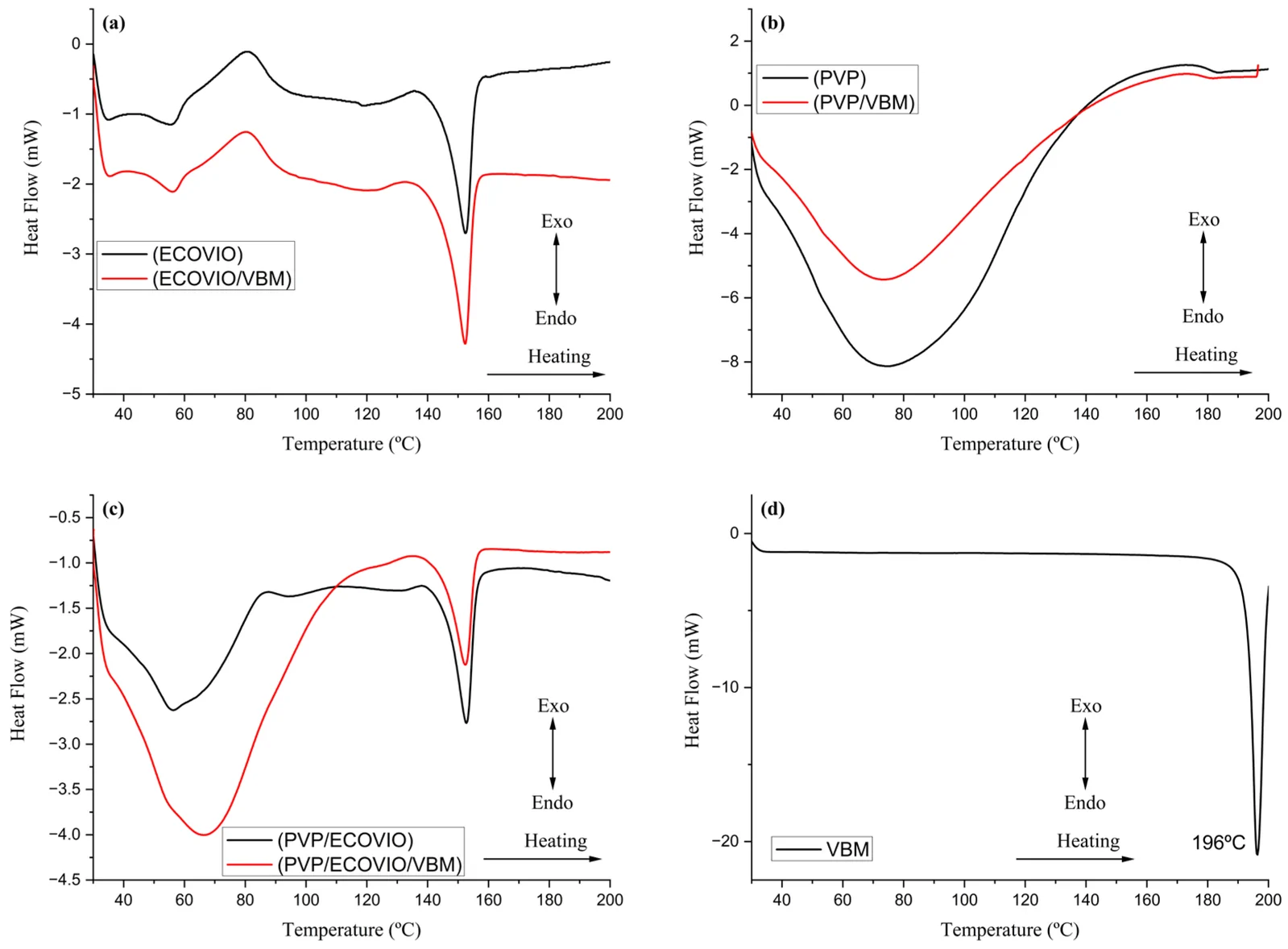
DSC curves. (**a**) ECOVIO^®^ and ECOVIO/VBM^®^; (**b**) PVP and PVP/VBM; (**c**) PVP/ECOVIO^®^ and PVP/ECOVIO^®^/VBM; (**d**) VBM.

DSC analysis complements the results of thermal analysis to verify the impact of drug incorporation into the membranes, as well as the interaction between the polymers used. Figure 5 shows the result of the DSC analysis for the electrospun membranes, as well as for the VBM.

The ECOVIO^®^ membrane presented three well-defined peaks in the DSC thermogram (Figure 5a). The endothermic peak at 57 °C refers to both the glass transition (T_g_) of PLA and the melting point (T_m_) of the crystalline phase of PBAT. The exothermic peak at 80 °C is associated with the recrystallization of PLA. Subsequently, the melting of PLA occurs at approximately 152 °C [29].

For the PVP membrane, a broad endothermic peak can be observed in Figure 5b up to 140 °C, characteristic of hygroscopic and amorphous substances [30]. The presence of ECOVIO^®^ and PVP peaks can be observed in the other membranes (Figure 5c). However, the peaks referring to T_g_ and T_m_ of ECOVIO^®^ up to 120 °C were superimposed on the PVP dehydration peak in the membranes containing the two polymers.

VBM is considered a crystalline drug and presents only one endothermic peak at 196 °C (Figure 5d), corresponding to the melting point [31]. It was not possible to observe the melting peak of VBM in any of the membranes, possibly due to the low concentration of VBM incorporated into membranes, and it might not be detected. The drug possibly incorporated into the membranes in an amorphous form is supported by the increase in VBM solubility during the In Vitro dissolution essay, which is discussed later.

#### 3.1.5. X-Ray Diffractometry

**Figure 6:**
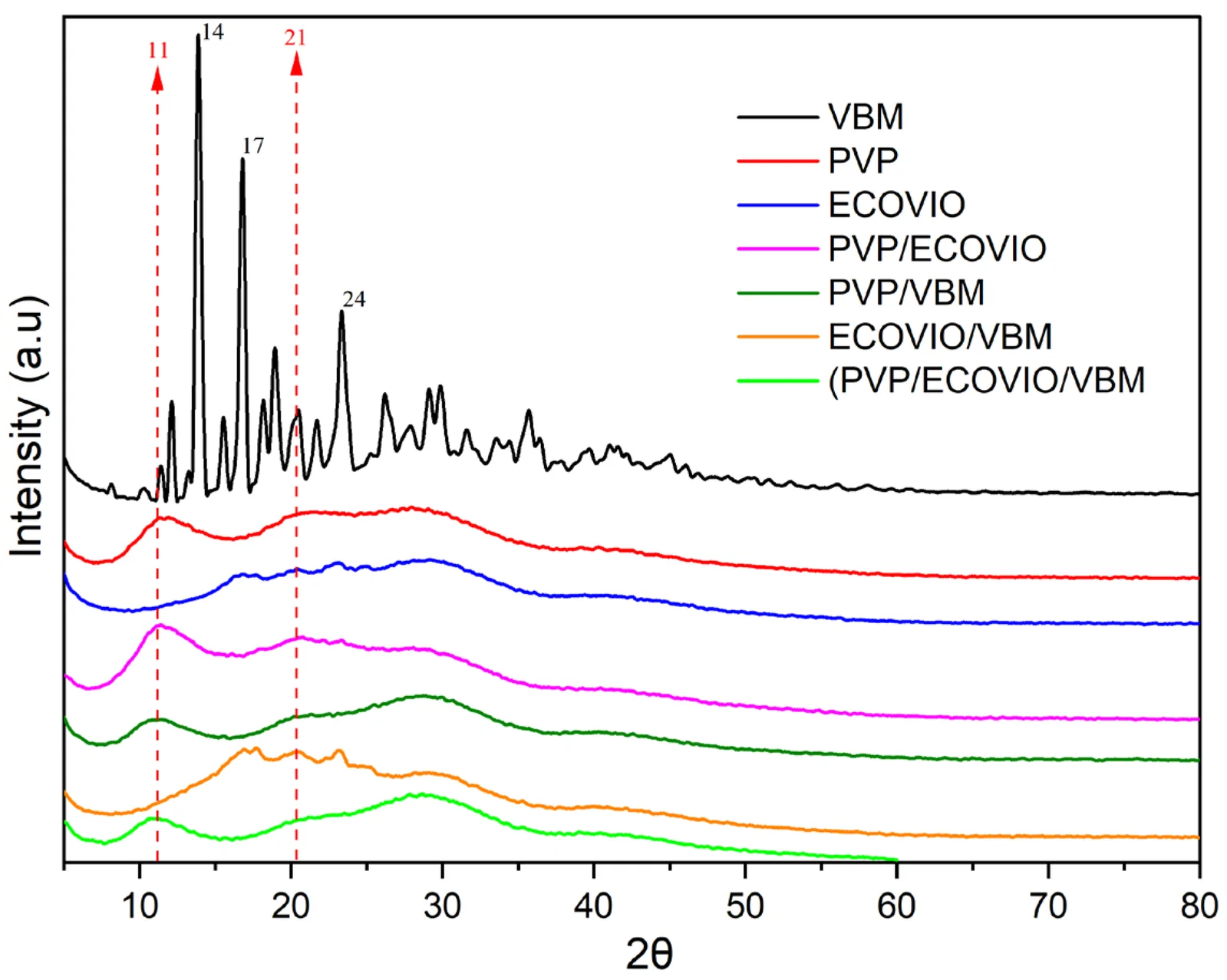
X-ray diffractogram for pure VBM and the membranes PVP, PVP/VBM, ECOVIO^®^, ECOVIO^®^/VBM, PVP/ECOVIO^®^ e PVP/ECOVIO^®^/VBM.

Figure 6 shows the X-ray diffraction spectra of the drug and the membranes PVP, PVP/VBM, ECOVIO^®^/VBM, PVP/ECOVIO^®^, and PVP/ECOVIO^®^/VBM. It can be observed from the X-ray spectra that all electrospun membranes present characteristics of semi-crystalline solids. The membrane (PVP) presents two broad peaks characteristic of the PVP polymer at 2θ = 11° and 21° [27], which can also be observed in the membranes PVP/VBM, PVP/ECOVIO^®^, and PVP/ECOVIO^®^/VBM, demonstrating the presence of PVP in the membranes.

In the X-ray diffractogram of the VBM, intense peaks are observed at 2θ 14°, 17°, and 24°, demonstrating high crystallinity and stability of the drug. However, in none of the electrospun membranes containing the drug were it possible to observe an increase in crystallinity. Similarly, electrospun with VBM presented the same crystallographic profile as that observed in the membranes that do not contain the drug, indicating that, just as in the DSC analysis, it may not have been detected. Although during detection sensitivity, the drug was possibly incorporated at an amorphous phase during the electrospinning process regarding the increased VBM solubility further discussed.

#### 3.1.6. VBM’s In Vitro Release

**Figure 7:**
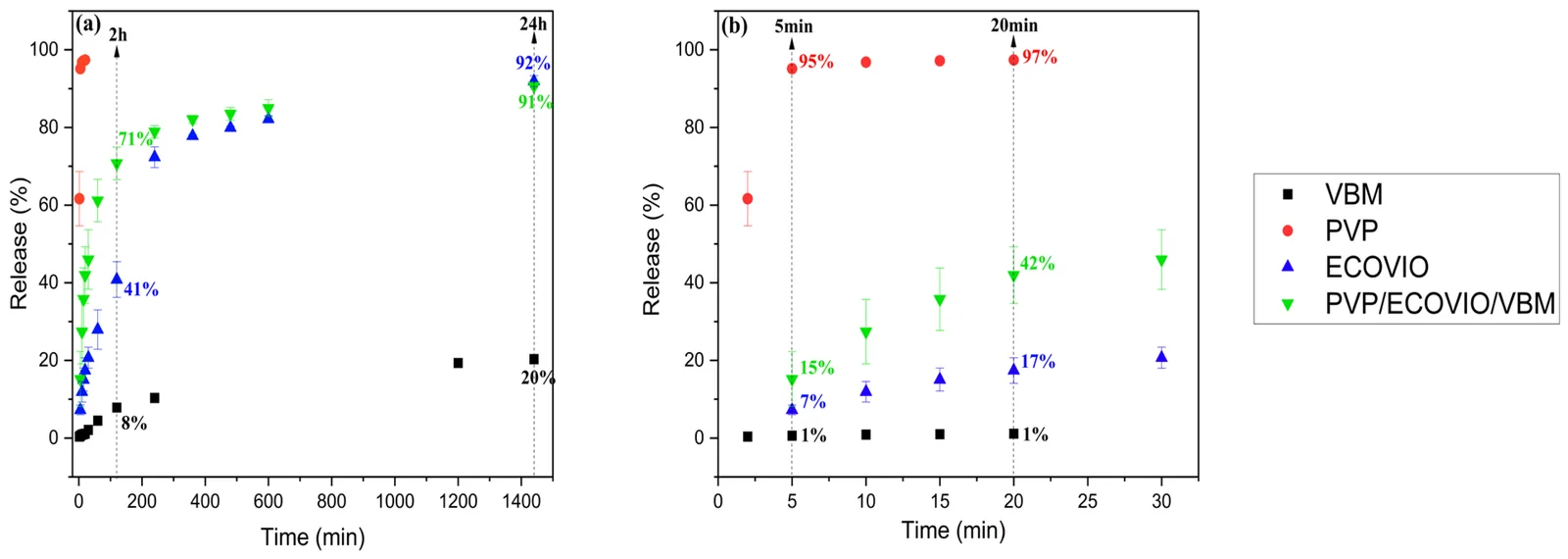
Drug solubilization and release profile of membranes PVP/VBM, ECOVIO^®^/VBM, and PVP/ECOVIO^®^/VBM carried out in triplicate for (**a**) 24 h and (**b**) 20 min. The dots indicate the arithmetic means, and the vertical bars represent the sample standard deviation (n = 3).

The VBM drug-release profile from the PVP/VBM, ECOVIO^®^/VBM, and PVP/ECOVIO^®^/VBM membranes and the drug solubility study are shown in Figure 7. The release study was carried out until complete solubilization of the drug from the membrane was reached, allowing the cumulative release percentages to be calculated based on the total amount of VBM initially incorporated into the electrospinning solutions.

Betamethasone valerate is soluble in ethanol (33 mg mL^−1^) and practically insoluble in water [32]. After 24 h of stirring at 100 rpm in phosphate buffer pH 5.7 maintained at a temperature of 32 °C, only 20% of the VBM mass was solubilized. When incorporated into a hydrophilic matrix (PVP/VBM membrane), the drug solubility increased by more than 90% in a period of 5 min. The release curve of the PVP/VBM membrane shows that in 5 min, approximately 95% of VBM was released into the medium, remaining constant at this concentration until 20 min of collection. Membranes electrospun with the PVP polymer tend to improve the solubility of hydrophobic drugs, since they aid in the dispersion of the drug [33].

For the ECOVIO^®^/VBM membrane, the drug release occurs in a more sustained manner. In 20 min, only 17% of VBM was released into the medium; in 2 h, it was possible to obtain 41% release, and in 24 h, approximately 92% of the drug was available in the medium. The slow release of VBM from this membrane is caused both by the hydrophobic characteristic of the polymers present in ECOVIO^®^ (PLA and PBAT) and by the characteristics of the drug itself. To obtain a low initial release and sustain it for a certain period, the incorporation of a hydrophobic drug in a hydrophobic matrix is what will achieve the desired result [33].

For the membrane PVP/ECOVIO^®^/VBM, it is possible to observe an initial release of 42% in 20 min, 71% in 2 h, and 91% in 24 h. The increase in the initial release of the biaxial membrane in relation to the membrane ECOVIO^®^/VBM is related to the PVP portion that releases the drug as soon as it comes into contact with the dissolution medium. The remainder of the release is related to the ECOVIO^®^ portion.

The release kinetics of VBM in the membranes PVP/VBM, ECOVIO^®^/VBM, and PVP/ECOVIO^®^/VBM were investigated using the mathematical models of Zero Order, First Order, Higuchi, Korsmeyer-Peppas, Hixson-Crowell, and Weibull through DDSolver 1.0 software. The study of release kinetics is essential for the quantitative evaluation of drug-release characteristics, as well as for comparing dissolution profiles. The DDSolver 1.0 software provides a variety of statistical parameters for the evaluation and definition of the best model fit that represents the obtained release data. The most used criteria for evaluation are the adjusted coefficient of determination (adjusted R^2^), Akaike information criterion (AIC), and the model selection criterion (MSC) [17].

Table 2 presents the results of the adjusted R^2^, AIC, and MSC values obtained for the models evaluated in the VBM releases from the membranes PVP/VBM, ECOVIO^®^/VBM, and PVP/ECOVIO^®^/VBM.

Evaluating the adjusted R^2^, AIC, and MSC values, the Weibull model presented the best fit among the evaluated models for describing the release profiles of VBM from the electrospun membranes.

The Weibull model is represented by Equation (1):

Mt/M∞ = 1 − exp [−((t − Ti)/a)b](1)

where Mt/M∞ is the fraction of drug released at time t, Ti is the lag time, a is the scale parameter associated with the time dependence of the process, and b is the shape parameter describing the curve profile.

The Weibull model provided the best fit for all membrane formulations, as evidenced by the higher adjusted R^2^ values and the more favorable AIC and MSC criteria (Table 3). The scale factor (a) reflects the time dependence of the release process, with lower values indicating faster drug release. Accordingly, the PVP/VBM membrane exhibited the lowest a value (0.56), consistent with its rapid release profile and the hydrophilic nature of PVP, which promotes fast dissolution of the polymer matrix and immediate drug availability. In contrast, the ECOVIO^®^/VBM membrane showed the highest value (57.97), indicating a slower release process associated with the hydrophobic characteristics of the PLA/PBAT blend. The biaxial membrane PVP/ECOVIO^®^/VBM presented an intermediate value (5.604), reflecting the combined action of the hydrophilic and hydrophobic fibers and corroborating the biphasic release profile observed experimentally.

The shape factor (b) was lower than 1 for all formulations, with values ranging from 0.13 to 0.73. According to the empirical interpretation of the Weibull model, b values below 1 indicate release curves characterized by an initially fast release rate followed by a progressive reduction over time. This behavior agrees with the experimental profiles obtained, particularly for the PVP-containing membranes, which exhibited an initial burst release phase followed by a slower release stage. Therefore, although the Weibull model should not be directly associated with a specific release mechanism, the fitted parameters provide a useful quantitative description of the distinct release behaviors promoted by the different membrane compositions.

It is important to emphasize that the Weibull equation is an empirical mathematical model widely used to fit dissolution and release data. Therefore, in the present study, the selection of the Weibull model is based solely on its superior statistical fit and should not be interpreted as evidence of a specific drug-release mechanism.

Although the release medium was selected to mimic certain physiological conditions of the skin surface, the present assay was designed exclusively to evaluate VBM release from the polymeric matrices. Consequently, the results cannot be directly correlated with skin penetration, skin retention, or therapeutic performance. Additional studies using Franz diffusion cells and biological skin models are required to investigate the topical delivery potential of these membranes.

## 4. Conclusions

A topical drug-delivery membrane based on ECOVIO^®^/PVP fibers was produced by biaxial electrospinning for the release of betamethasone valerate. The membrane produced presented uniform fibers that did not have beads, were thermally stable, and had amorphous characteristics. The electrospinning of the ECOVIO^®^ polymer solution, together with the PVP polymer solution in the biaxial mode, suggests that ECOVIO^®^ makes the PVP fibers less susceptible to moisture, observed by the SEM technique, by the lower occurrence of coalescence in the biaxial membrane, in relation to the PVP membranes. The FTIR technique demonstrated chemical compatibility between the drug and the PVP and ECOVIO^®^ polymers due to the absence of displacement of the bands of the main functional group characteristic of each molecule. TGA demonstrated a possible increase in the thermal stability of the polymers and the drug due to the increase in the degradation temperature of the molecules. And the XRD technique showed a possible amorphization of the VBM when incorporated into the membranes due to the absence of crystallinity in the spectra obtained. Through the previous in vitro release analysis, it was possible to observe an increase in the solubility of VBM when incorporated into the PVP membrane. The release of the drug from the membrane PVP/ECOVIO^®^/VBM demonstrated an initial rapid release probably originating from the PVP portion, followed by a sustained release due to ECOVIO^®^. The Weibull model provided the best statistical fit to the experimental release data and was therefore considered the most suitable empirical model for describing the in vitro release profiles obtained in this study. Thus, the results demonstrate the feasibility of producing electrospun membranes that promote a biphasic in vitro release profile of betamethasone valerate, characterized by an initial rapid phase followed by a sustained phase. However, the present study was limited to physicochemical characterization and in vitro drug release evaluation. Therefore, additional studies using Franz diffusion cells and biological skin models are required before conclusions can be drawn regarding skin permeation, skin retention, or potential therapeutic performance.

Although the present study demonstrates the feasibility of producing biaxially electrospun membranes with distinct in vitro release profiles, additional investigations are still required to assess their suitability for topical applications. Important functional and mechanical properties, including tensile strength, elasticity, thickness, porosity, wettability, moisture vapor permeability, and moisture resistance, were not evaluated in the current work and should be addressed in future studies.

## 5. Patents

A patent application resulting from the work reported in this manuscript has been filed by Universidade Estadual do Oeste do Paraná (Unioeste). The authors Édina Liara Schons, Diogo José Salamanca da Silva, Douglas Cardoso Dragunski, and Josiane Caetano are listed as inventors. The patent application is currently pending.

## Figures and Tables

**Table 1:** Composition of the membranes produced for the study.

Membrane	PVP Membrane		ECOVIO^®^ Membrane	
	% PVP (w v^−1^)	% VBM (w w^−1^)	% ECOVIO^®^ (w v^−1^)	% VBM (w w^−1^)
PVP	8	0	0	0
ECOVIO^®^	0	0	20	0
PVP/ECOVIO^®^	8	0	20	0
PVP/VBM	8	1	0	0
ECOVIO^®^/VBM	0	0	20	1
PVP/ECOVIO^®^/VBM	8	1	20	1

**Table 2:** Kinetic models applied to the release of VBM from membranes.

Membrane	Kinetic Model	Adjusted R^2^	AIC	MSC
PVP/VBM	Zero Order	−8.38	47.16	−2.57
	First Order	0.96	20.45	2.77
	Higuchi	−1.85	41.13	−1.36
	Korsmeyer-Peppas	0.59	32.47	0.37
	Hixson-Crowell	−0.38	37.26	−0.59
	Weibull	1.00	−7.39	8.34
ECOVIO^®^/VBM	Zero Order	0.098	113.80	−0.056
	First Order	0.94	79.49	2.64
	Higuchi	0.84	92.63	1.65
	Korsmeyer-Peppas	0.91	43.62	2.17
	Hixson-Crowell	0.87	89.17	1.94
	Weibull	0.98	67.76	3.72
PVP/ECOVIO^®^/VBM	Zero Order	−2.24	121.96	−1.28
	First Order	0.70	93.22	1.11
	Higuchi	−0.09	108.42	−0.16
	Korsmeyer-Peppas	0.88	84.01	1.88
	Hixson-Crowell	−0.36	110.79	−0.53
	Weibull	0.99	56.21	4.13

PVP—Polyvinylpyrrolidone; VBM—Betamethasone Valerate; R^2—^Determination Coefficient; AIC—Akaike information criterion; MSC—model selection criterion.

**Table 3:** Kinetic parameters for the Weibull model.

Membrane	Scale Factor (a)	Shape Factor (b)	Adjusted R^2^	AIC	MSC
PVP/VBM	0.56	0.13	1.00	−7.39	8.34
ECOVIO^®^/VBM	57.97	0.73	0.98	67.76	3.72
PVP/ECOVIO^®^/VBM	5.604	0.37	0.99	56.21	4.13

PVP—Polyvinylpyrrolidone; VBM—Betamethasone Valerate; R^2—^Determination Coefficient; AIC—Akaike information criterion; MSC—model selection criterion.

## Data Availability

Data is contained within the article. The original contributions presented in this study are included in the article/Supplementary Material. Further inquiries can be directed at the corresponding authors.

## Supplementary Materials

The following supporting information can be downloaded at: https://www.mdpi.com/article/10.3390/polym18212725/s1, Figure S1: VBM’s Calibration Curve; Figure S2: VBM’s UV-Vis Absorption Spectrum for concentrations of 0.5, 5.0, 10.0, 15.0, and 20.0 mg L^−1^.
