# Supplementary material for: Biaxial Electrospinning of Poly(lactic acid)/Poly(butylene adipate-co-terephthalate) Blend and Polyvinylpyrrolidone Membranes for Topical Delivery of Betamethasone Valerate
Source: Polymers (Basel). 2026 Nov 2;18(21):2725. doi: 10.3390/polym18212725 (PMC13766003; doi:10.3390/polym18212725)
Supplement: Supplementary file 1 [file polymers-18-02725-s001.zip › polymers-4480654-supplementary.pdf]

Article

Biaxial Electrospinning of Poly(lactic acid)/Poly(butylene adipate-co-terephthalate) Blend and Polyvinylpyrrolidone Membranes for Topical Delivery of Betamethasone Valerate

Édina Liara Schons [1], Diogo José Salamanca [1], Gabriel Nardi Fraga [2], Douglas Cardoso Dragunski [1] and Josiane Caetano [1,*]

[1] Master Program in Chemistry, State University of West Paraná, 645, Rua da Faculdade, Toledo 85903-000, Paraná, Brazil; edina.schons@gmail.com (É.L.S.); douglas.dragunski@unioeste.br (D.C.D.)

[2] Department of Chemistry, State University of Maringá, 5790, Av. Colombo, Maringá 87020-900, Paraná, Brazil; gabriel.fraga@unioeste.br

* Correspondence: josiane.dragunski@unioeste.br

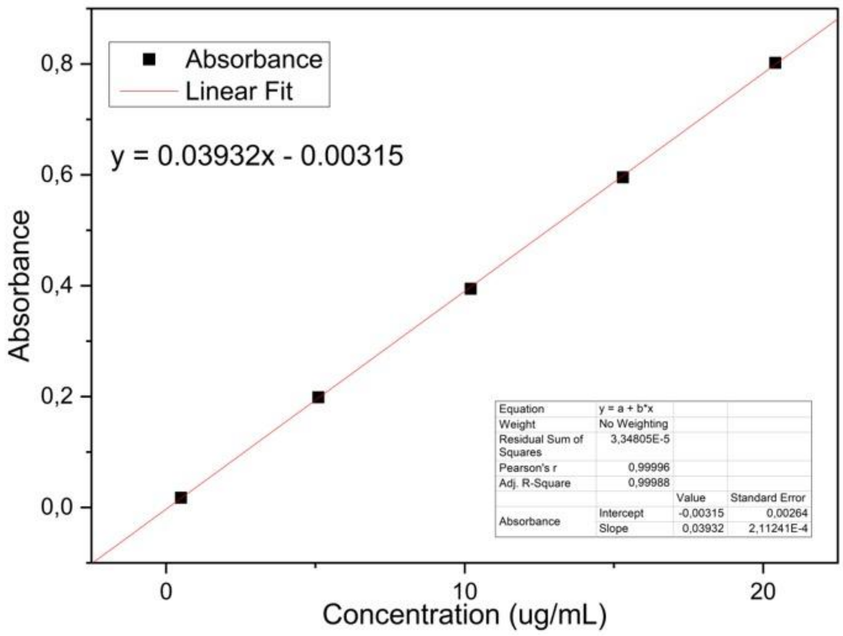

Figure S1. VBM's Calibration Curve

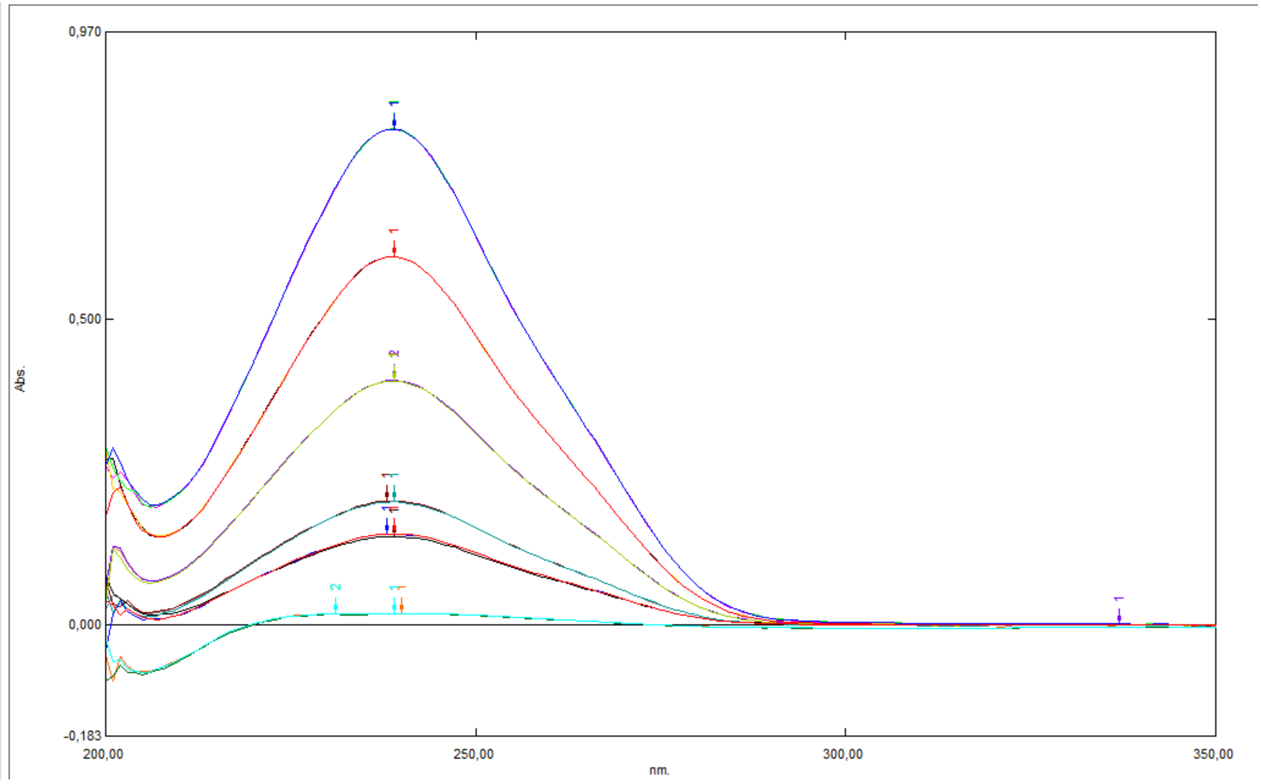

Figure S2. VBM's UV-Vis Absorption Spectrum for concentrations of 0.5, 5.0, 10.0, 15.0, and 20.0 mg L^{-1}
